# Munc13 mediates klotho-inhibitable diacylglycerol-stimulated exocytotic insertion of pre-docked TRPC6 vesicles

**DOI:** 10.1371/journal.pone.0229799

**Published:** 2020-03-05

**Authors:** Jian Xie, Sung-Wan An, Xin Jin, Yuan Gui, Chou-Long Huang

**Affiliations:** Division of Nephrology, Department of Internal Medicine, Carver College of Medicine, University of Iowa, Iowa City, Iowa, United States of America; Indiana University School of Medicine, UNITED STATES

## Abstract

α-Klotho is a type 1 transmembrane protein that exhibits aging suppression function. The large amino-terminal extracellular domain of α-klotho is shed as soluble klotho (sKlotho) and functions as a circulating cardioprotective hormone. Diacylglycerol (DAG)-activated calcium-permeable TRPC6 channel plays a critical role in stress-induced cardiac remodeling. DAG activates TRPC6 by acting directly on the channel to increase its activity and by stimulation of channel exocytosis. sKlotho protects the heart by inhibiting DAG stimulation of TRPC6 exocytosis. How DAG stimulates TRPC6 exocytosis and thereby inhibition by sKlotho are unknown. Using a compound that directly activates TRPC6 without affecting channel exocytosis, we validate that sKlotho selectively blocks DAG stimulation of channel exocytosis. We further show that DAG stimulates exocytosis of TRPC6-containing vesicles pre-docked to the plasma membrane. Mnuc13 family proteins play important roles in the proper assembly of SNARE proteins and priming the vesicle competent for fusion. We show that DAG stimulates TRPC6 exocytosis by targeting to the C1 domain of Munc13-2. The results provide fresh insights into the molecular mechanism by which DAG regulates vesicle fusion and how sKlotho protects the heart against injury.

## Introduction

α-Klotho is a type 1 transmembrane protein predominantly produced in the kidney, choroid plexuses of the brain, parathyroid gland, and to a lesser extend in several other tissues [1]. Mice homozygous for hypomorphic klotho allele (*kl*/*kl*) exhibit phenotypes resembling premature human aging [1]. Conversely, overexpression of α-klotho transgene in mice results in extended lifespan [2]. In humans, single nucleotide polymorphisms have been identified in the klotho gene that correlate with reduced longevity and the pathophysiology of age-related disorders such as osteoporosis, coronary artery disease, and stroke [3–6].

Membranous α-klotho associates with fibroblast growth factor (FGF) receptors to form co-receptors for the ligand FGF23 [7, 8]. FGF23 is a bone-derived circulating hormone that lowers serum phosphate levels by increasing renal phosphate excretion, suppressing 1,25-dihyroxyvitamin D synthesis, and decreasing gastrointestinal phosphate absorption [9]. α-Klotho-deficient mice have severe hyperphosphatemia due to defects in the klotho-FGF23-vitamin D regulatory axis. Phosphate retention is pivotal for growth retardation and premature death of α-klotho-deficient mice. Dietary phosphate restriction rescues growth defects and premature death of the mice [9, 10].

The full-length membranous α-klotho is comprised of a large extracellular domain of 952 amino acids (human klotho), a transmembrane-spanning segment, a short 11 amino acids intracellular carboxyl terminus [1]. The extracellular domain can be cleaved by metalloproteases and shed into the systemic circulation, urine and cerebrospinal fluid as soluble klotho (sKlotho) [11, 12]. Multiple lines of evidence indicate that sKlotho functions as circulating endocrine hormone or local autocrine/paracrine factor [13]. Membranous α-klotho is not normally expressed in myocardium. Circulating sKlotho inhibits transient receptor potential channel TRPC6-mediated calcium signaling in the heart thereby protecting against stress-induced cardiac injury [14, 15]. Reduction in circulating levels of sKlotho underlies aggravated cardiac hypertrophy in mouse models of chronic kidney disease (CKD) [16] and probably in patients with CKD.

Rise in the myocardial intracellular Ca^2+^ at the T tube-sarcoplasmic reticulum junction during the systolic phase initiates cardiac contraction. Abnormal Ca^2+^ signaling occurring in perinuclear or subsarcolemmal compartments and in both systolic and diastolic phases leads to pathological cardiac remodeling [13]. Calcium influx through TRPC channels including TRPC6 activates cardiac genes involved in hypertrophic growth via the calcineurin-nuclear factor of activated T cells (NFAT) cascade [17, 18]. TRPC6 is pivotal because it contains NFAT-responsive elements in its promoter, which amplifies and sustains cardiac hypertrophic gene expression through a feed-forward circuit [17, 18]. We have shown that sKlotho protects against stress-induced cardiac hypertrophy by inhibiting growth factor-stimulated phosphoinositide 3-kinase (PI3K)-dependent exocytosis of TRPC6 channels [14, 15].

TRPC6 is a receptor-activated channel stimulated by diacylglycerol (DAG) released from phospholipase C (PLC)-mediated hydrolysis of phosphatidylinositol-4,5-bisphophate (PIP_2_) [19]. DAG activates TRPC6 via dual mechanisms: direct channel activation and stimulation of exocytosis of the channel [14, 15, 20, 21]. How DAG stimulates TRPC6 exocytosis and thereby its inhibition by sKlotho are unknown. In this study, we provide evidence that TRPC6-containing vesicles are pre-docked to the plasmalemma and that DAG stimulates fusion of the pre-docked vesicles by acting on Munc13-2.

## Materials and methods

### Electrophysiological recordings of TRPC6 currents

Established human embryonic kidney 293 (HEK293) cells obtained from the American Type Culture Collection (ATCC® CRL-1573™) were transfected with plasmid encoding C-terminal GFP tagged TRPC6. When indicated siRNA for human Munc13-2 (from Santa Cruz Biotechnology, Cat # sc-42022) or control oligonucleotides and/or plasmids encoding mouse *ub*Munc13-2 or Munc13-4 were cotransfected.

TRPC6 currents were recorded from HEK293 cells expressing recombinant TRPC6 (identified by green fluorescence) with or without indicated modulators as previously described [14, 15]. When indicated, cells expressing TRPC6 were treated sKlotho (100 pM) in serum-containing medium overnight. Currents were recorded by voltage-clamp in ruptured whole-cell mode with an Axopatch 200B patch-clamp amplifier and Pulse software (Molecular Devices, Sunnyvale, CA, USA). Cells were held at 0 mV membrane potential and stimulated with repetitive ascending ramp pulse from −100 mV to +100 mV for 400 ms every 10 s. The pipette and bath solution contained (in mM) 140 CsCl, 1 MgCl2 1.5 CaCl2, 2 ATP-Mg, 5 EGTA, 10 HEPES (pH 7.2 with CsOH) and 140 NaCl, 0.5 EGTA, 10 HEPES, 10 glucose, 10 mannitol (pH 7.4 with NaOH), respectively. In experiments in Fig 3C, pipette solution contained TTX A chain, BAPTA (1,2-bis(o-aminophenoxy)ethane-*N*,*N*,*N′*,*N′*-tetraacetic acid), and ZnCl_2_ as indicated. The resistance of electrodes containing pipette solution was 1.5–3 MΩ. TRPC6 currents were activated by bath application of oleoyl-acetyl-glycerol (OAG; 100 μM), low pass filtered, at 2 kHz and sampled every 0.1 ms (10 kHz). At the end of experiments, the bath solution was replaced by a solution containing nonpermeant cation N-methyl-d-glucamine (NMDG) to assess TRPC6-mediated inward currents. Data acquisition and analysis were performed using the pClamp v.9.2 program (Molecular Devices) and Prism v.3.0 software (GraphPad Software, La Jolla, CA, USA).

### Statistical analysis

Statistical comparison was made between control and experimental groups conducted during the same time period. Each experiment was repeated at least 3 times with similar results. Data are presented as means ± SEM. Statistical comparison between two groups of data were made using two-tailed unpaired Student’s t-test. Multiple comparisons were determined using one-way analysis of variance followed by Tukey’s multiple comparison tests.

## Results

**Fig 1:**
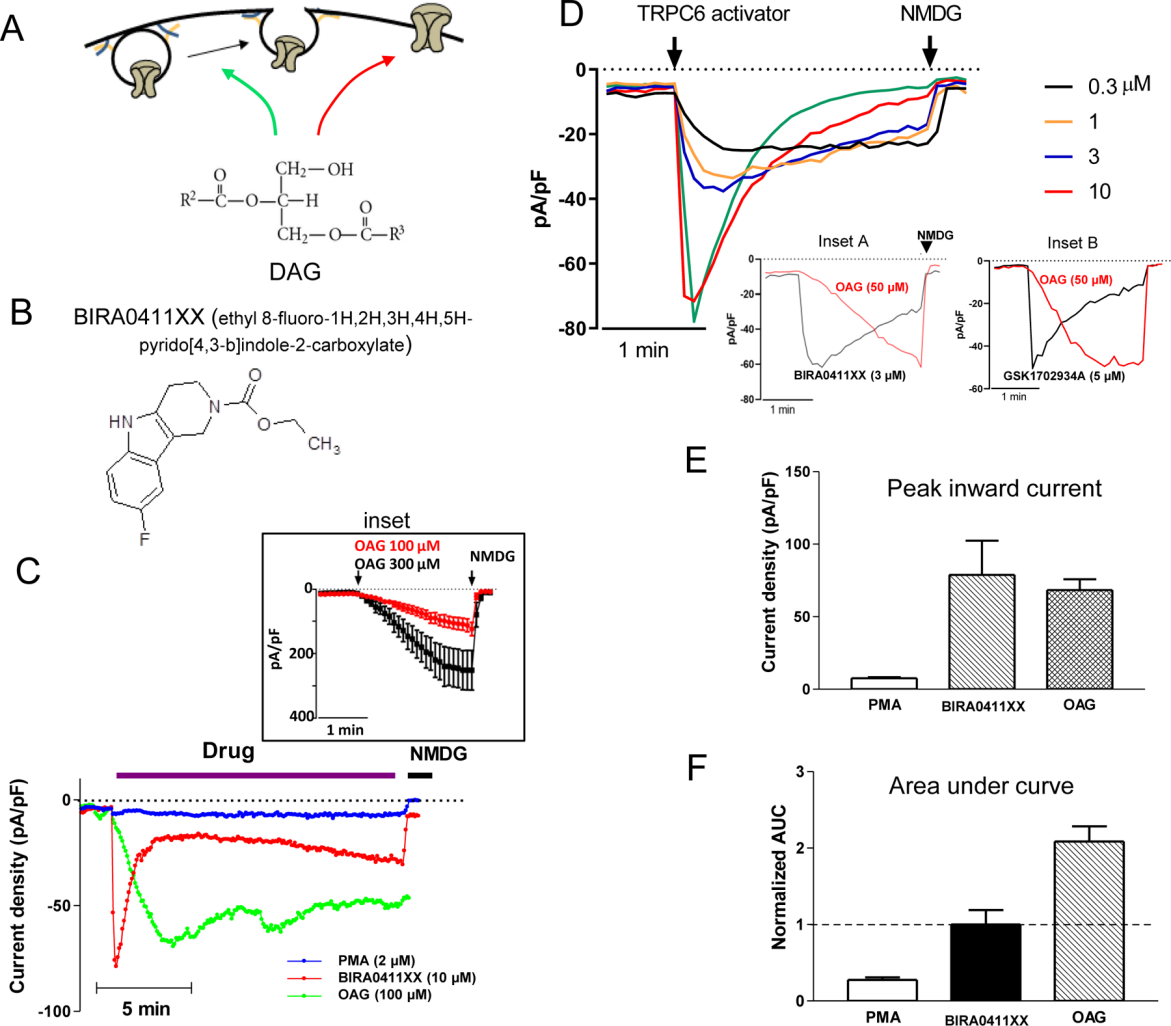
Diacylglycerol (DAG) stimulates TRPC6 exocytosis as well as directly activates the channel. (A) Diagram illustrating dual mechanisms of activation of TRPC6 by DAG. (B) Chemical structure of TRPC6 channel activator BIRA0411XX. (C) Time course of TRPC6 activation by DAG and by BIRA0411XX. TRPC6-mediated currents were recorded in whole-cell mode from HEK cells expressing recombinant TRPC6. After baseline recording, membrane permeable DAG analog (oleoly-acetyl-glycerol, OAG), BIRA0411XX or PMA (phorbol myristate acetate) was applied extracellularly. NMDG (*N*-Methyl-D-glucamine) was added at the end of experiment. Inset shows dose-dependent activation of TRPC6 by OAG (100 and 300 μM). Shown are representative tracings of current density (pA/pF) at -100 mV. Inset shows dose-dependent relationship of OAG activation of TRPC6. (D) Dose-response activation of TRPC6 current by BIRA0411XX. Inset A and B shows representative time course of activation by BIRA0411XX and by GSK1702934A, respectively, versus 100 μM OAG. (E) Bar graph shows mean ± SEM of peak inward current density from multiple experiments as in panel C. (F) Mean ± SEM of area under curve (AUC) from multiple experiments as in panel C. Data are normalized relative to BIRA0411XX.

Originally, DAG is identified as a direct activator of TRPC6 [19]. The effect is believed due to direct action of DAG on the channel, independently of the effect of DAG to stimulate protein kinase C (PKC). Subsequent studies showed that DAG also stimulates exocytosis of TRPC6 [14, 15, 20, 21]. To further validate the notion of dual mechanisms of action of TRPC6 by DAG (Fig 1A), we employed a recently available compound, BIRA0411XX, believed to directly activate TRPC6 (Fig 1B) [22]. In HEK cells expressing recombinant TRPC6 channels, extracellular application of BIRA0411XX (at the maximally active concentration) increased TRPC6 currents in 5 sec reaching a peak in 10–20 sec (Fig 1C). BIRA0411XX-activated current decayed over ~1 min to a steady-state lower level. BIRA0411XX had no effect on un-transfected HEK cells (7.2 ±1.5 pA/pF vs 8.3 ± 2.4 pA/pF, without and with BIRA0411XX; not significant, n = 5 each).

Application of DAG dose-dependently increased TRPC6 current, and the current increases follow a significantly slower time course (Fig 1C, inset). Fig 1C compares the kinetics of TRPC6 current activated by the maximal concentration of BIRA0411XX (10 μM; see Fig 1D) with the kinetics of currents activated by a submaximal concentration of DAG (100 μM) that gives rise to the same level of peak current density. As shown, DAG-induced current reached the peak in ~3 min, and the current remained elevated over ~15 min. Since the effect of DAG on TRPC6 is not mediated by PKC, we used PKC activator phorbol ester (PMA) as a negative control and found it had no effects on cells expressing TRPC6. The differences in the kinetics between BIRA0411XX and DAG remained when a lower concentration of activator was used (Fig 1D, 1D inset A). The differences in kinetics between BIRA0411XX and DAG were substantiated by using another known direct TRPC6 activator, GSK1702934A (Fig 1D, inset A and inset B). The differences in the mode of action between DAG and BIRA0411XX are further illustrated by the area-under-curve (AUC) of current density. We found that AUC of current density is greater for DAG than for BIRA0411XX using the concentration of compounds that give equal peak current density (Fig 1E and 1F). The results are consistent with the notion that DAG activates an additional process, that is exocytosis of the channel.

**Fig 2:**
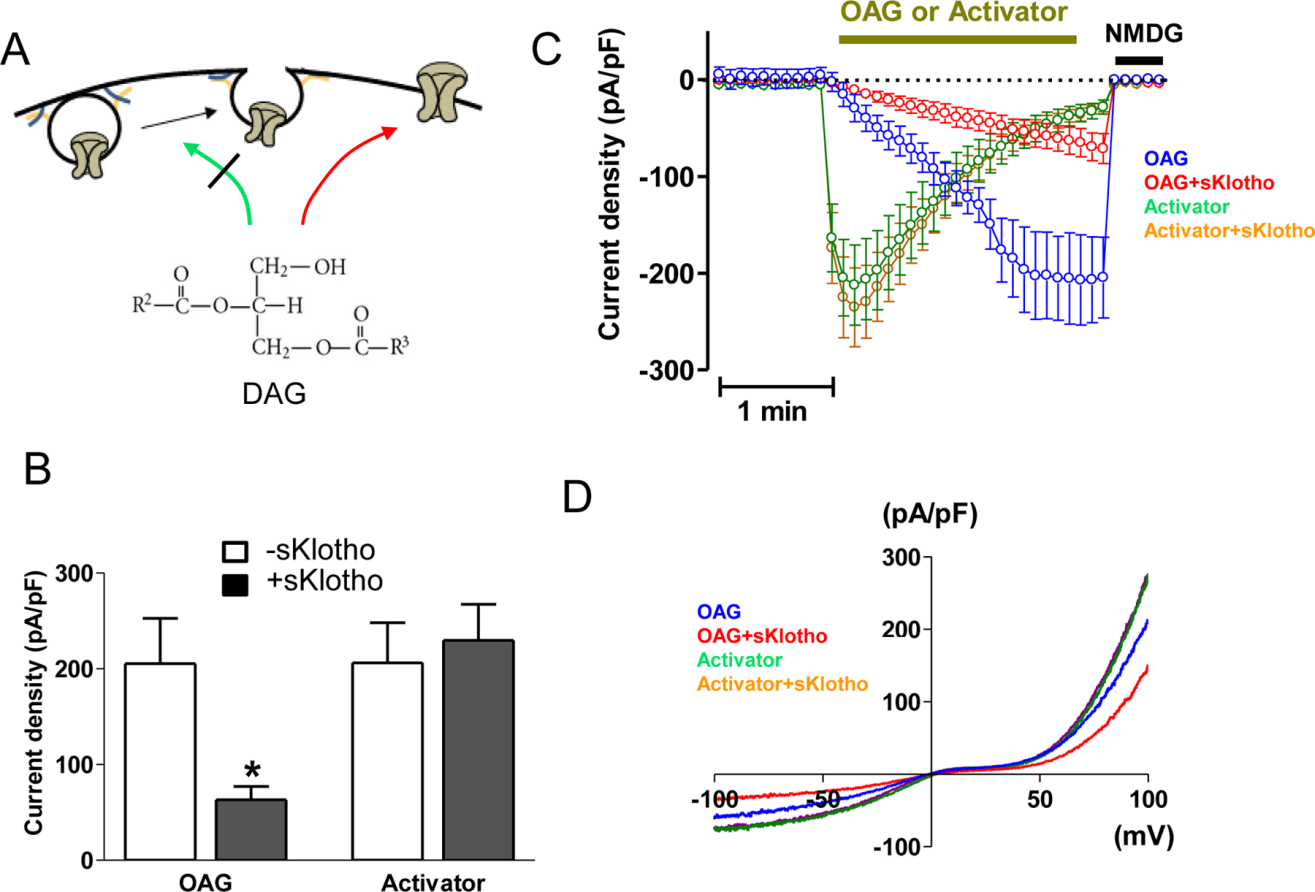
Soluble klotho selectively blocks DAG-stimulated TRPC6 exocytosis. (A) Diagram illustrating sKlotho selectively blocks exocytosis. (B-D) Effect of sKlotho on TRPC6 currents stimulated by BIRA0411XX or by OAG. HEK cells expressing recombinant TRPC6 channels were treated with sKlotho or vehicle overnight. TRPC6 currents were activated by BIRA0411XX (labeled as “activator”) or OAG. Panel B shows representative current-voltage (I-V) relationship curve. Panel C shows time course of TRPC6 currents. Panel D shows mean ± SEM (n = 6–8 each) of peak inward current density from multiple experiments. Please note that the I-V curve of “activator + sKlotho” (orange line) essentially overlaps with that of “activator” alone (green line).

In our previous studies, we found that sKlotho and exocytosis blocker tetanus toxin (TTX) each partially blocks DAG-stimulated TRPC6 current and that their effects are not additive [14, 15]. The results support the notion that sKlotho inhibits DAG stimulation of exocytosis (Fig 2A). To further differentiate the two processes induced by DAG and validate the notion that sKlotho does not affect direct channel activation, we took the advantage of the fact that direct channel activator does not stimulate TRPC6 exocytosis. We found that sKlotho partially inhibited DAG-stimulated TRPC6 while had no effect on BIRA0411XX-induced TRPC6 current (Fig 2B). sKlotho did not alter the time course and current-voltage relationship of BIRA0411XX-induced TRPC6 current (Fig 2C and 2D), further supporting that sKlotho selectively affects channel exocytosis, but does not inhibit channel directly.

**Fig 3:**
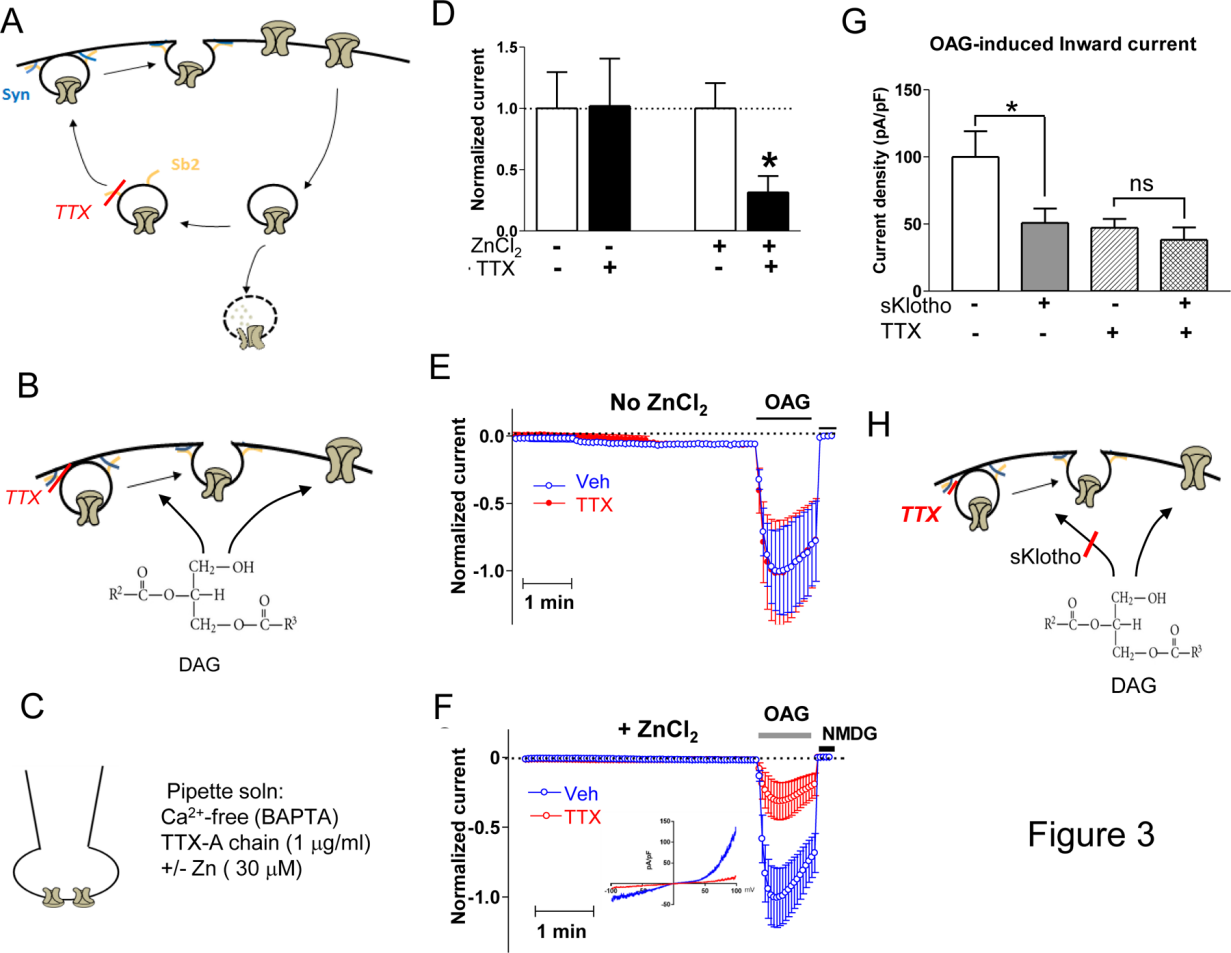
Soluble klotho blocks DAG stimulation of exocytotic insertion of pre-docked TRPC6 vesicles. (A, B) Tetanus toxin (TTX) acts on synaptobrevin (“Sb2” for synaptobrevin-2) to decrease surface expression of TRPC6 channels. The effect may occur at the level of recycling vesicles (panel A) or on pre-docked vesicles (panel B). (C) Diagram illustrating pipette solution used in whole-cell patch-clamp recording. (D) In the presence of ZnCl_2_, TTX-A chain (applied in patch pipette) decreased DAG-stimulated TRPC6 current. TTX-A chain has no effect in the absence of ZnCl_2_. Current density (at -100 mV) normalized to recording without ZnCl_2_ and without TTX (first bar from left). Bar graph shows mean ± SEM, n = 6–8. * indicates p< 0.01 vs no TTX. (E, F) Time course of recordings showing effect of TTX in the absence of ZnCl_2_ (panel E) or presence of ZnCl_2_ (panel F). Inset in panel F shows I-V curves with Veh vs TTX. (G) Relationship between effect of TTX and soluble klotho on OAG-stimulated TRPC6 current. Bar graph shows mean ± SEM, n = 6–8. * indicates p< 0.01 as indicated. ns, statistically no significant. (H) Diagram illustrating site of action of TTX and sKlotho.

TTX cleaves *v*-SNARE (soluble N-ethylmaleimide-sensitive [NSF] factor attachment receptor) synaptobrevin-2 to interfere with vesicle trafficking. The effect can occur to the intracellular trafficking vesicles such as recycling vesicles (Fig 3A) or de novo formed vesicles en route from endoplasmic reticulum-Golgi to the plasmalemma (not shown). It may also act on vesicles already docked to the plasmalemma in which SNARE proteins are loosely assembled but remain accessible to the toxin (Fig 3B). In our previous studies, we preincubated cells with TTX for 2–4 hours to allow time for toxin to enter cells [14, 15]. These experiments using long preincubation however cannot differentiate the effect of TTX on intracellular vesicles from its effect on vesicles already tethered to plasmalemma. To differentiate between the two effects, we applied the enzymatically active subunit of TTX (A chain) to the whole-cell patch pipette (Fig 3C). This experimental approach allows the toxin to diffuse into the intracellular space within a few minutes after formation of ruptured whole-cell configuration. The intracellular Ca^2+^ concentration is buffered to sub-physiological levels by Ca^2+^ chelator BAPTA to arrest recycling and forward trafficking of vesicles. The pipette solution contains with or without ZnCl_2_, an essential cofactor for TTX’s enzymatic activity [23]. Under the experimental condition, we found that TTX (directly delivered via pipette) partially blocked DAG-induced TRPC6 current (Fig 3D–3F). The effect of TTX required ZnCl_2_, indicating it is specific effect of the toxin. The results support the notion that DAG stimulates fusion of TRPC6 vesicles pre-docked to the plasmalemma. The effect of inhibition of TRPC6 by the pipette-delivered TTX is not additive to the inhibition exerted by sKlotho (Fig 3G and 3H), indicating they act on the same pathway.

**Fig 4:**
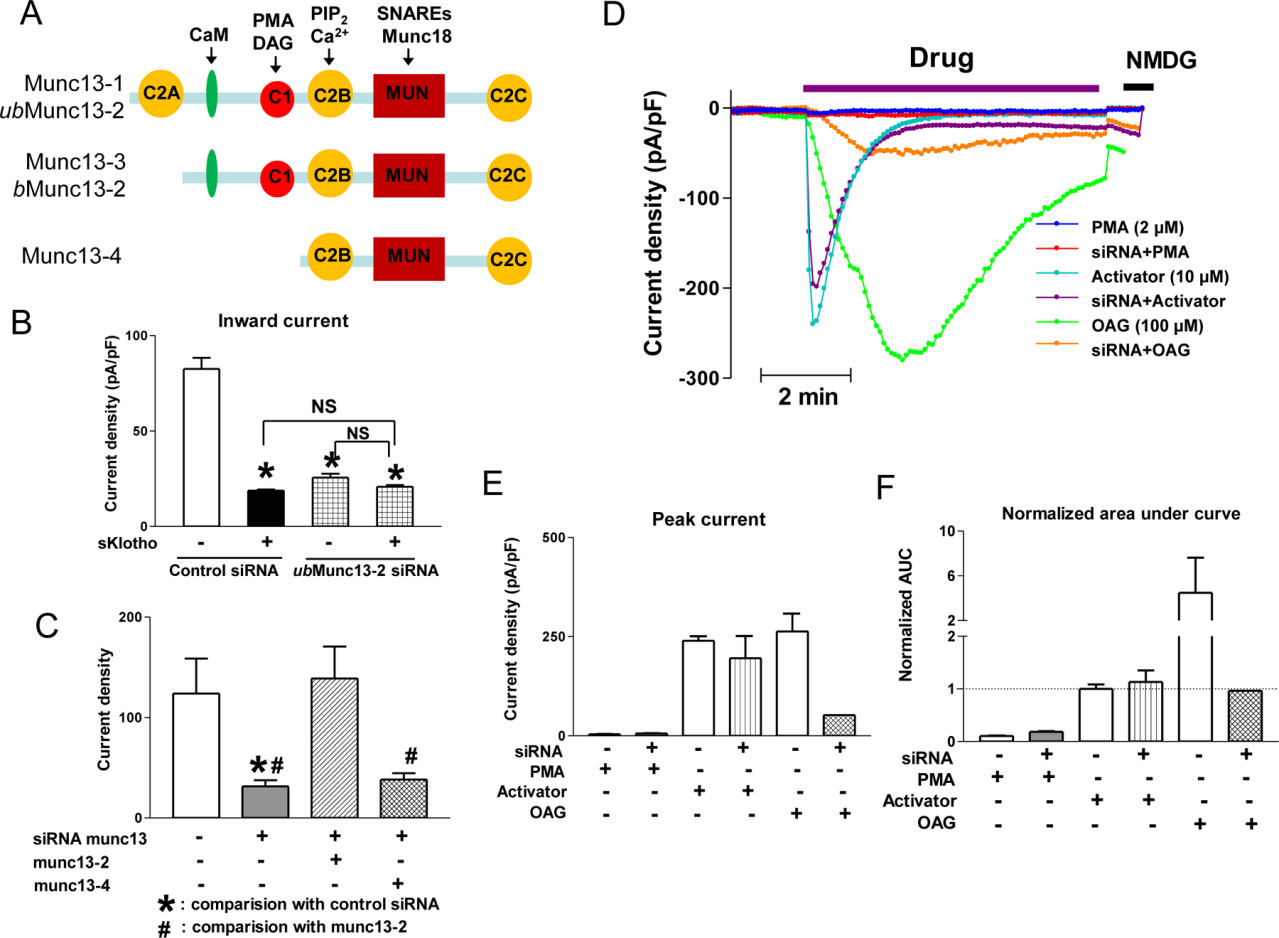
DAG stimulates TRPC6 exocytosis acting at Munc13-2. (A) Functional domains of Munc13 family proteins. *ub*Munc13-2; ubiquitously expressed Munc13-2; *b*Munc13-2, brain-specific Munc13-2. C1, DAG (and phorbol ester)-binding domain; C2B, calcium and phospholipid-binding domain; C2A and C2C, homology to C2B, but Ca^2+^-independent; MUN domain, conserved domain for binding Munc18 and SNARE proteins. Domain for calmodulin (CaM) binding (green long oval) is also shown. (B) Effect of Munc13-2 knockdown on inhibition of TRPC6 currents by sKlotho. HEK cells were transfected with GFP-TRPC6 and siRNA against human *ub*Munc13-2 isoforms or control oligos for ~36 hr before ruptured whole-cell recording of TRPC6 currents. Cells were incubated with sKlotho or vehicle overnight before recording. TRPC6 currents were activated by OAG. Bar graph shows mean ± SEM of peak inward current density at -100 mV, n = 6–8 each. * indicates p< 0.01 vs control (control siRNA and no sKlotho). ns, statistically no significant between indicated. (C) Effect of re-expressing recombinant mouse ubiquitous Munc13-2 or Munc13-4 after Munc13-2 knockdown. (D) Effects of Munc13 knockdown on TRPC6 channels stimulated by OAG or BIRA0411XX (labeled as “activator”). Representative time course tracings of experiments are shown. PMA does not activate TRPC6 and was used as a negative control. (E) Mean ± SEM of peak inward current density of experiments performed in panel D. (F) Mean ± SEM of normalized area under curve (AUC) of inward currents of experiments in panel D.

We next investigated the mechanism by which DAG stimulates exocytosis. Among proteins involved in the vesicular trafficking, Munc13 family proteins contain a DAG (and phorbol ester)-binding C1 domain. There are 4 mammalian Munc13 genes coding for 5 Munc13 proteins [24] (Fig 4A). Munc13-1 and Munc13-3 are predominantly expressed in brain. Munc13-2 gene has two promoters which produce a longer ubiquitously expressed Munc13-2 (*ub*Munc13-2) and a shorter brain-specific Munc13-2 (*b*Munc13-2), respectively. Munc13-4 is the smallest Munc13 family member widely expressed in peripheral tissues. We focused on *ub*Munc13-2 and Munc13-4 because of their peripheral distribution. Among them, we considered *ub*Munc13-2 is the most likely candidate target for DAG because the existence of DAG-binding C1 domain. Indeed, we found that knocking down *ub*Munc13-2 by siRNA partially decreased the DAG-induced TRPC6 current (Fig 4B). The effect of *ub*Munc13-2 knockdown is not additive to the effect of sKlotho, supporting that sKlotho inhibits TRPC6 exocytosis mediated by *ub*Munc13-2. To further support the role of *ub*Munc13-2, re-expression of mouse *ub*Munc13-2 in the knockdown cells rescued DAG-induced TRPC6 current (Fig 4C). In contrast, re-expression of Munc13-4 which lacks the DAG-binding C1 domain failed to rescue the effect of knockdown. The specificity of Munc13 knockdown on vesicle fusion is further authenticated by its lack of effect on BIRA0411XX-induced TRPC6 current (Fig 4D–4F). As shown, while knocking down Munc13-2 by siRNA largely inhibited DAG-induced TRPC6 current, it had no effect on TRPC6 current induced by BIRA0411XX. The specificity of TRPC6 current is evident by the response to stimulation by DAG, but not by PMA. The differential effect of Munc13-2 knockdown on DAG-induced vs BIRA0411XX-induced TRPC6 is apparent when analyzed either by the peak current density or AUC of current.

**Fig 5:**
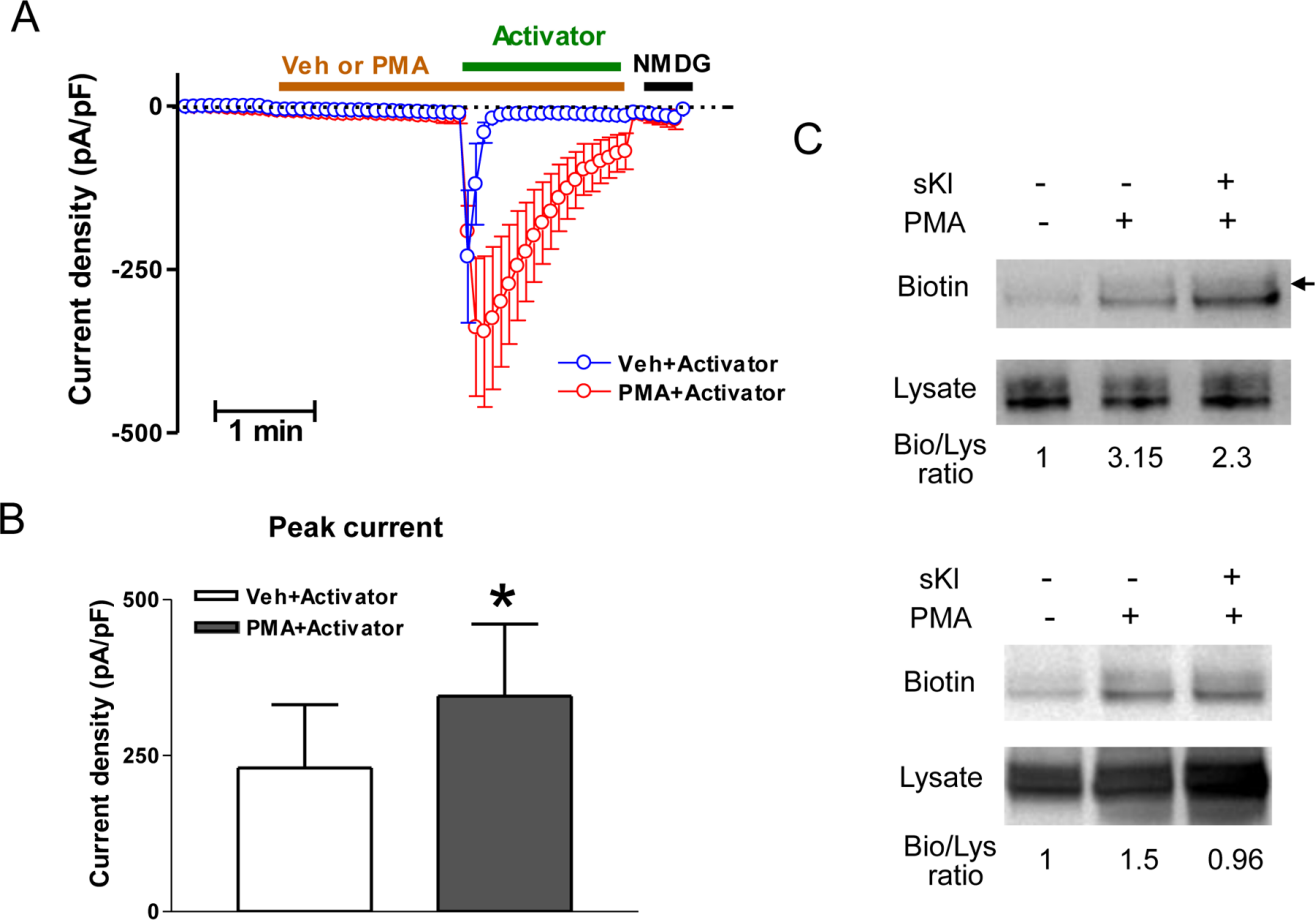
Additional evidence supporting that DAG stimulates TRPC6 exocytosis acting at Munc13. (A) Effect of PMA vs vehicle on TRPC6 activated by BIRA0411XX. HEK cells expressing TRPC6 were treated with PMA or vehicle (DMSO) for ~2 min before application of BIRA0411XX (labeled as “activator”). (B) Mean ± SEM of peak inward current density of experiments performed in panel A. (C) PMA increases cell-surface expression of TRPC6, which is inhibited by sKlotho. Cell surface abundance of Flag-tagged TRPC6 expression in HEK cells were analyzed by biotinylation. Abundance of biotinylated TRPC6 protein detected by anti-Flag antibody (labeled “Biotin”; analyzed by densitometry) was divided by the total TRPC6 abundance in lysates (labeled “Lysate”), and the ratios (labeled “Bio/Lys ratio”) were normalized to the setting without PMA, without sKlotho (first lane from left). Shown are two separate experiments with similar results. Numerical values indicate normalized ratios. Arrow indicates glycosylated TRPC6. Please notes that gycosylation is normally heterogenous that may occur in only 1 or 2 subunits of tetrameric channels such as TRPC6.

Unlike the effect of direct channel activation on TRPC6 that is exerted by DAG but not PMA, both PMA and DAG activate Munc13 by binding to its C1 domain [24]. We therefore used PMA to selectively activate channel exocytosis (via Munc13) and BIRA0411XX to selectively activate channel without causing exocytosis. These approaches allow us to distinguish between the two mechanisms of DAG and to further validate the mechanism of DAG stimulation of exocytosis via Munc13. As shown, cells expressing TRPC6 were treated first with PMA or vehicle to target Munc13, then followed by application of BIRA0411XX to activate TRPC6 current from channels at the cell surface (Fig 5A and 5B). No TRPC6 current was observed during PMA pretreatment, confirming that PMA does not share the effect of DAG to directly activate the channel. PMA treatment yet enhanced the TRPC6 response to BIRA0411XX. This is due to that PMA activates Munc13 to stimulate channel exocytosis. To support this notion, PMA treatment increased cell-surface expression of TRPC6 assessed by surface biotinylation (Fig 5C). Overall, these results provide strong support for the notion that DAG acts on Munc13 to stimulate TRPC6 exocytosis. In these experiments, the effect of DAG to stimulate exocytosis is recapitulated by PMA. In contrast, the direct effect of DAG to activate TRPC6 is not recapitulated by PMA.

## Discussion

In the mechanism of membrane fusion, SNARE proteins must assemble into *trans*-SNARE complexes to provide the machinery and force for vesicle fusion [24]. The SNARE complexes are formed of four α-helix domains, 1 each from synaptobrevin and syntaxin, and 2 from SNAP-25. The rate-limiting step in the assembly process is the association of the syntaxin, which in the basal state is in closed form incapable of interacting with other SNARE proteins. Munc18 and Munc13 play important roles in the proper assembly of *trans*-SNARE complexes [24, 25]. Munc18 binds syntaxin to form Munc18-closed syntaxin complexes. Munc13 interacts with Munc18-syntaxin complex through the conserved MUN domain, which leads to opening of syntaxin for assembling with SNAP-25 and synaptobrevin. These processes mediated by Munc13 tether vesicles to the plasmalemma, rendering them competent for fusion.

We have previously shown that sKlotho protects the heart by inhibiting TRPC6 channel exocytosis stimulated by DAG [14, 15]. The molecular mechanism for these processes remains largely unknown. In the present study, we show that sKlotho inhibits exocytosis of TRPC6 vesicles pre-docked (tethered) to the plasmalemma. This conclusion is based on experiments by direct application of tetanus toxin in the patch pipette in the condition where only membrane-tethered vesicles are available for fusion. Under the condition, sKlotho and tetanus toxin both partially inhibit TRPC6 and their effects are not additive. We further elucidate the mechanism by which DAG stimulates TRPC6 exocytosis. We show that knocking down endogenous Munc13-2 abolishes DAG stimulation of TRPC6, which can be rescued by re-introduction of recombinant Munc13-2, not by Munc13-4 lacking the DAG-binding C1 domain. Thus, DAG acts on Munc13-2 to lead to membrane fusion of pre-docked TRPC6-containing vesicles. Additional important finding of our present study includes using a direct channel activator to solidify the notion that DAG activates TRPC6 via dual mechanisms. We show that sKlotho, known to inhibit DAG-induced channel exocytosis but not direct channel activation, does not affect TRPC6 current induced by direct channel activator.

DAG has been implicated in mediating the stimulus-induced sustained secretory granule release, which occurs either in response to stimulation of PLC-coupled G-protein-coupled receptors or through non-receptor mechanisms [26, 27]. Two previous studies reported that Munc13-1 is involved in this process. Kang et al showed Munc13-1 is required for glucose-induced sustained release of insulin in pancreatic β cells [26]. Bauer et al also reported that Munc13-1 is required for histamine-induced sustained neurotransmitter secretion in bovine chromaffin cells [27]. Our current study shows that Munc13-2 is essential for DAG stimulation of TRPC6 exocytosis. Our report supports the notion that Munc13 proteins that contain DAG binding domain play important roles in DAG-mediated membrane fusion. The ubiquitously expressed Munc13-2 is the likely Munc13 protein mediates the process in cells lacking Munc13-1.

Pre-docking of vesicles to target membranes is essential for fast release of vesicular contents. In the neuronal synapses, the presynaptic neurotransmitter-containing vesicles are pre-docked to synaptic active zones in the axonal terminals. Upon arrival of action potential and membrane depolarization, Ca^2+^ enters cells through voltage-gated Ca^2+^ channels. Ca^2+^ ions bind to synaptotagmin to trigger vesicle fusion and neurotransmitter release. Pre-docking of vesicles to synaptic active zones allows neurotransmitter release to occur in less than milliseconds. What then are biological reasons for vesicles pre-docking for exocytosis processes that occur in minutes or longer, such as TRPC6 exocytosis? We suggest that pre-docking functions as a mechanism of spatial compartmentalization of TRPC6. In the systolic phase of cardiac cycle, the intracellular [Ca^2+^] rises to ≥10 μM to trigger cardiac contraction. Pathological cardiac remodeling and hypertrophy are also triggered by Ca^2+^, however at levels only slightly above the basal resting [Ca^2+^]. Spatial compartmentalization is essential for cells to distinguish between the two processes. Physiological contractile Ca^2+^ release occur at T-tube and sarcoplasmic reticulum junction whereas pathological Ca^2+^ elevation occurs at either nuclear/perinuclear or specialized plasma membrane domains, such as lipid rafts/caveolae [13]. Association of TRPC6 channels in lipid rafts/caveolae has been demonstrated [28]. We have shown that sKlotho specifically targets to lipid rafts and regulates PI3K-Akt signaling cascade in lipid rafts, but not in the non-raft membranes [15]. PI3K-Akt signaling is involved in TRPC6 channel exocytosis [14, 15, 21]. The selective distribution of TRPC6 to raft membranes and selectivity of sKlotho to TRPC6 in the rafts explain why sKlotho protects against TRPC6-mediated cardiac injury but does not affect physiological cardiac contraction. We hypothesize that the mechanism(s) that mediates selective distribution of TRPC6 to lipid rafts also underlies pre-docking, which ensures signaling occurring at the right place. Future studies will investigate the mechanism(s) that mediates TRPC6 targeting to lipid rafts and pre-docking.

## Supporting information

S1 File (PDF)
