# Supplementary material for: Munc13 mediates klotho-inhibitable diacylglycerol-stimulated exocytotic insertion of pre-docked TRPC6 vesicles
Source: PLoS One. 2020 Mar 5;15(3):e0229799. doi: 10.1371/journal.pone.0229799 (PMC7058344; doi:10.1371/journal.pone.0229799)

Original gel for Figure 5C, Experiment 1. “S” and “SD” indicates cells were cultured overnight in serum-containing and serum-deprived medium respectively before PMA and/or sKl treatment.

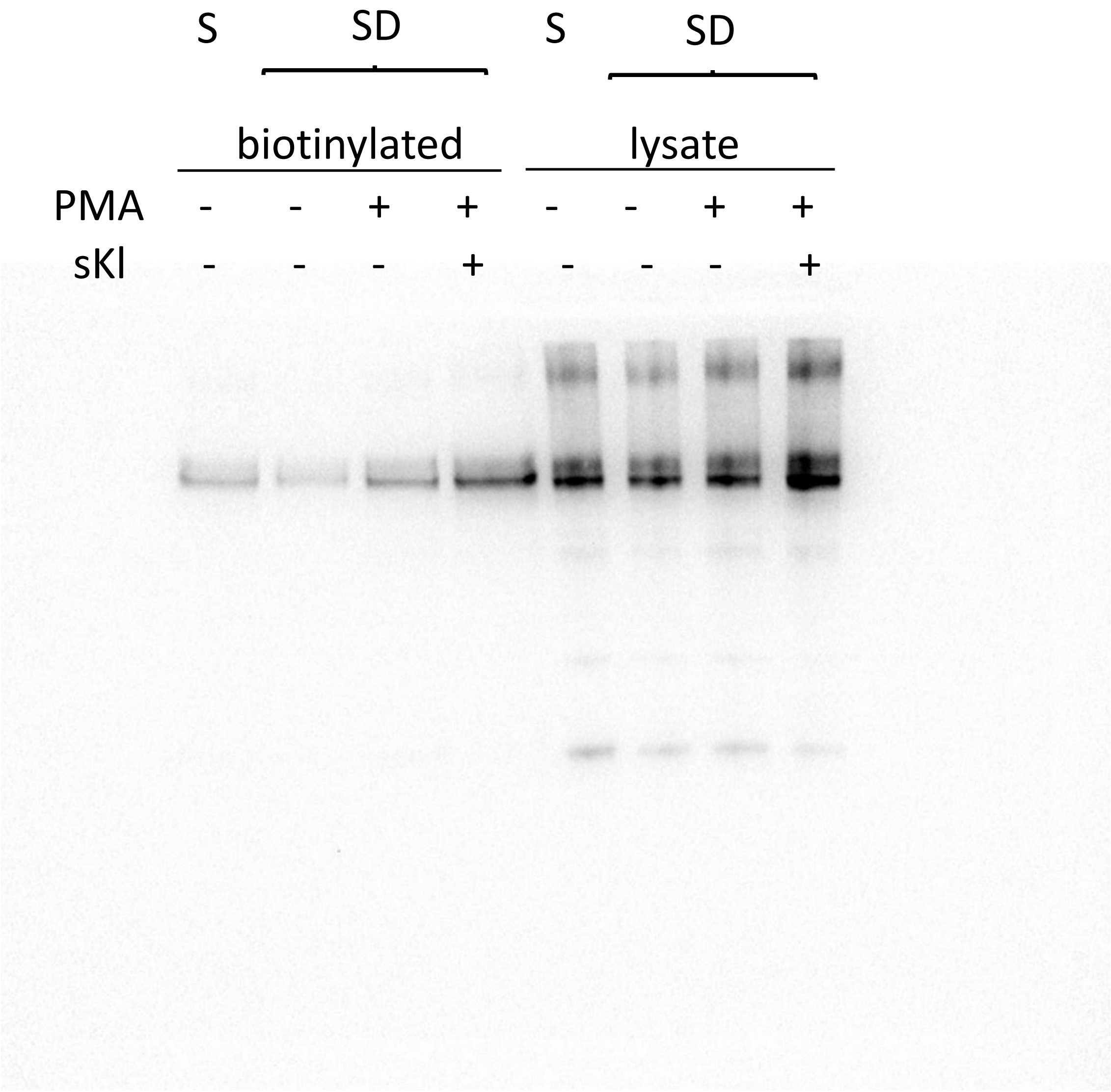

Original gel for Figure 5C, Experiment 2.
“S” and “SD” indicates cells were cultured overnight in serum-containing and serum-deprived medium respectively before PMA and/or sKl treatment.

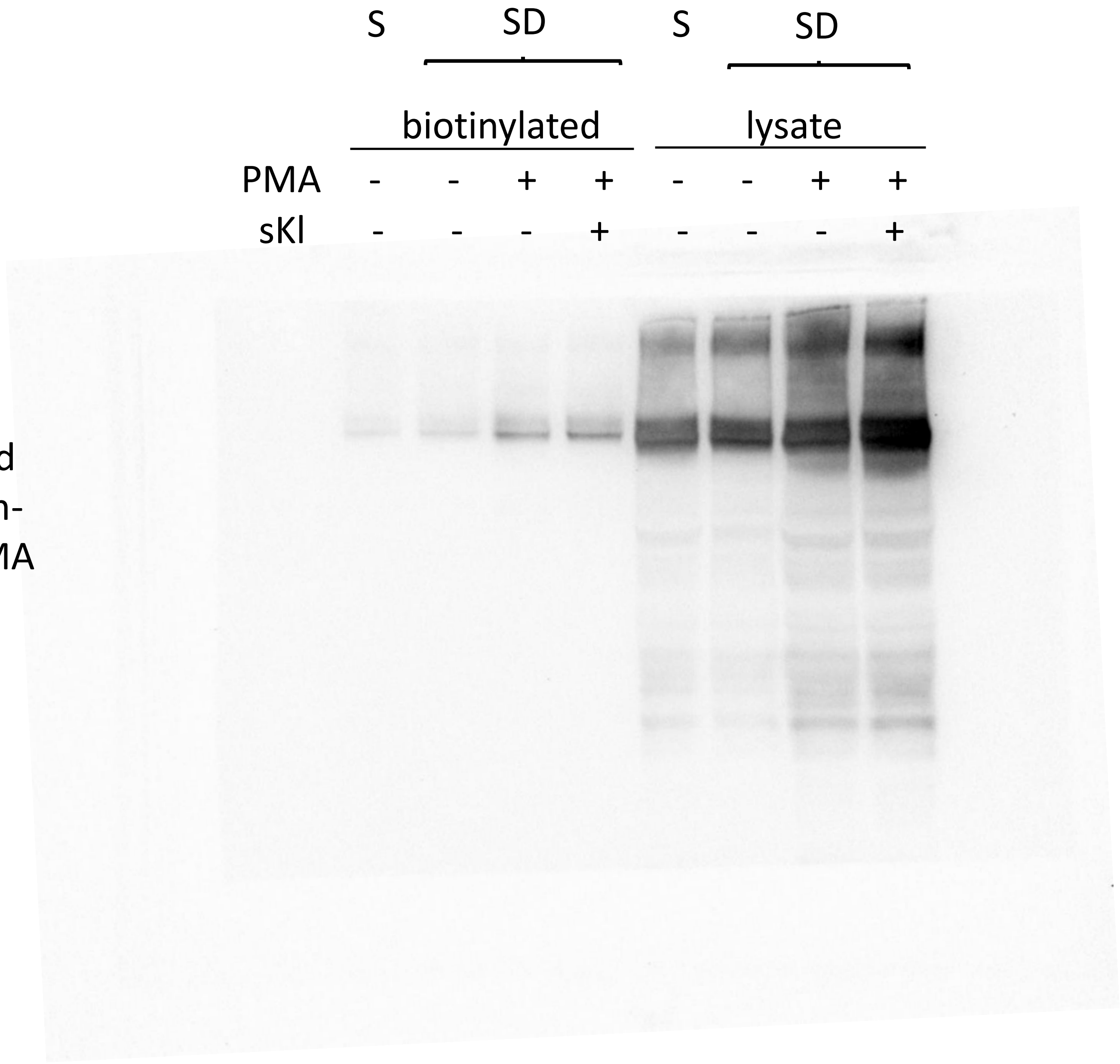

Supplement: S1 File — (PDF) [file pone.0229799.s001.pdf]
